# Airway management in pediatrics: improving safety

**DOI:** 10.1007/s00540-024-03428-z

**Published:** 2024-11-18

**Authors:** Lea Zimmermann, Federica Maiellare, Francis Veyckemans, Alexander Fuchs, Tommaso Scquizzato, Thomas Riva, Nicola Disma

**Affiliations:** 1 Department of Anesthesiology and Pain Medicine, Inselspital, Bern University Hospital, University of Bern, Bern, Switzerland; 2 Unit for Research in Anesthesia, IRCCS Istituto Giannina Gaslini, Via G. Gaslini 5, 16100 Genoa, Italy; 3 UCLouvain Medical School, Brussels, Belgium; 4 Department of Anaesthesia and Intensive Care, IRCCS San Raffaele Scientific Institute, Milan, Italy

**Keywords:** Airway, Pediatric, Anesthesia, Intubation, Complications

## Abstract

Airway management in children poses unique challenges due to the different anatomy, physiology, and pathophysiology across the pediatric age span. The recently published joint *European Society of Anaesthesiology and Intensive Care*-*British Journal of Anaesthesia* (ESAIC-BJA) neonatal and infant airway management guidelines provide recommendations and suggestions to support clinicians in deciding the best strategy. These guidelines represent a framework with the most recent and up-to-date evidence, from the initial assessment to the management of normal and difficult airways up to the extubation phase. However, such guidelines have intrinsic limitations due to the lack of supporting evidence in various fields of airway management. Pediatric institutions should adopt guidelines after careful internal review according to the local circumstances, including caseload, equipment and expertise. The current narrative review focused on providing references and practical tips on pediatric airway management, which is still not completely elucidated. Moreover, the authors put particular emphasis on the influence of human factors on the overall success of tracheal intubation, the incidence of complications, and the outcomes for patients.

**Supplementary Information:**

The online version contains supplementary material available at 10.1007/s00540-024-03428-z.

## Introduction

Managing the pediatric airway is fundamental in pediatric anesthesia, critical care, and emergency medicine. Children have anatomical and physiological characteristics that require specialized approaches to ensure their safety. Successful airway management results from a delicate balance between understanding the pediatric unique airway anatomy, technical skills, rapidly evolving medical knowledge, and the availability of new technologies. Moreover, children's metabolic rates are high, and their oxygen reserves are small, which means that any compromise in their airway can rapidly lead to hypoxemia and its consequences. This puts a premium on the efficiency in securing the airway. The margin for error is small, and the consequences of delays or mistakes can be critical.

The recently published guidelines on neonatal and infant airway management [1, 2] highlighted point-by-point the current level of evidence on the management of normal, expected, and unexpected difficult airways, from initial assessment to extubation. However, in several fields, the level of evidence was still low, and experts’ opinion was obtained through the usual Delphi consensus. The lack of scientific or clinical evidence in some recommendations in these guidelines should serve as a base for future trials to fill these knowledge gaps. Moreover, airway management, especially in young patients with complex diseases, has historically been based more on personal skills and experience than on published evidence. Moving ‘from art to evidence’ represents a paradigm shift that should improve patient safety. Based on the current evidence and experts’ practice, we aim to provide some practical tips with insights into the most controversial areas of airway management in children and discuss the impact of human factors on success and performance.

## Methods

We conducted a comprehensive literature review on pediatric airway management; LZ and FM independently searched the databases on Ovid Medline, PubMed, Web of Science, and Embase. Search terms included: “preparation for airway management,” “preoperative assessment,” “difficult airway prediction in children,” “pediatric or infant difficult airway,” “apneic oxygenation,” “supplemental oxygen,” “oxygenation,” “anesthesia induction and neuromuscular blocking agents,” “video laryngoscopy for pediatric airway management,” “pediatric emergency front-of-neck-access,” “pediatric or infant eFONA,” and “pediatric or infant cricothyrotomy or tracheostomy.” We placed no date restriction on the literature search; we determined that 3512 articles were relevant from the abstracts. After screening these abstracts, 38 studies were considered appropriate to the current review.

### Pre-operative assessment

Medical history, especially previous airway management, and physical examination are mandatory as they provide valuable information about potential risks and appropriate strategies to ensure safe and effective airway management. The presence of medical conditions (e.g., infection) or anatomical features (whether congenital or acquired) can significantly influence the approach to airway management.

Several tools have been described to predict a difficult airway, including the most recent application of Artificial Intelligence, Machine Learning [3], and preoperative airway ultrasound [4]. However, they present with two major limitations: the first is that an airway identified as non-difficult by clinical assessment and sophisticated tools can be difficult after induction of anesthesia or after unsuccessful initial management has resulted in edema, bleeding, or spasm. Second, none of the existing tools have been able to guide the practitioner on the best management strategy when a difficult airway has been identified.

Based on the above considerations, clinical evaluation and, if available, expert opinion remain the most reliable methods for predicting a difficult airway. Table 1 summarizes the essential and mandatory physical assessment to systematically evaluate a child’s airway.

**Table 1:** Physical assessment to predict a difficult airway management

Anatomy	Assessment	Difficult face mask ventilation	Difficult laryngoscopy
Mouth opening	Restricted mouth opening Mouth opening is restricted when its height is less than three fingers of the child's hand	±	++
Soft tissues	Presence of oral, pharyngeal or neck masses whether congenital or acquired (infection, tumor)	++	++
Thyro-mental distance	Reduced distance between chin and thyroid cartilage with extended head To measure the thyro-mental distance, use three fingers of the child’s hand	±	++
Shape and size of maxilla, mandibula, and ears	Mandibular or midface hypoplasia; facial asymmetry; external ear anomalies	++	++
Flexion and extension of neck	Reduced neck movement or neck at risk for subluxation of vertebrae	±	++
Mallampati score in children > 5 years		±	+

It is also essential to recognize that the airway characteristics in some syndromes can evolve, either spontaneously or following corrective surgery (e.g., mandibular or maxillar osteotomy), and impact the difficulty of intubation. Some syndromes worsen, making intubation more and more challenging, while others improve progressively, facilitating the process. For instance, in patients with Pierre Robin syndrome, micrognathia and glossoptosis become less severe over time, making intubation easier. Conversely, in conditions like Treacher Collins syndrome, airway management becomes increasingly more difficult as the craniofacial anomalies tend to worsen with age.

In the same way, mucopolysaccharidoses (MPS) can cause progressive airway obstruction as glycosaminoglycans accumulate in tissues, making intubation increasingly difficult as the disease progresses, even after enzymatic treatment or bone marrow transplantation [5]. Closely monitoring these patients' evolving anatomical and physiological characteristics is crucial to plan timely and appropriate interventions. Managing airways in children with complex syndromes requires a multidisciplinary and personalized approach, considering the specific needs and potential changes in the patient's condition over time. Table 2 summarizes the changes in some syndromes over time.

**Table 2:** Impact of aging on syndromes associated with difficult airway management

Generally improves with ages	Worsen with ages
Pierre Robin sequence (micrognathia—jaw size increases)	Treacher Collins syndrome (micrognathia, small mouth, funnel-shaped larynx)
Goldenhar syndrome (asymmetrical micrognathia—jaw size increases)	Apert (midface anomalies, cervical fusion)
	Hunter and Hurler syndromes (mucopolysaccharides in tongue and larynx, infiltration of larynx and trachea which becomes tortuous)
	Beckwith–Wiedemann syndrome (macroglossia)
	Freeman–Sheldon syndrome (circumoral fibrosis and microstomia)
	Fibrodysplasia ossificans progressiva
	Klippel–Feil Syndrome (absence or fusion of cervical vertebrae

**Fig. 1:**
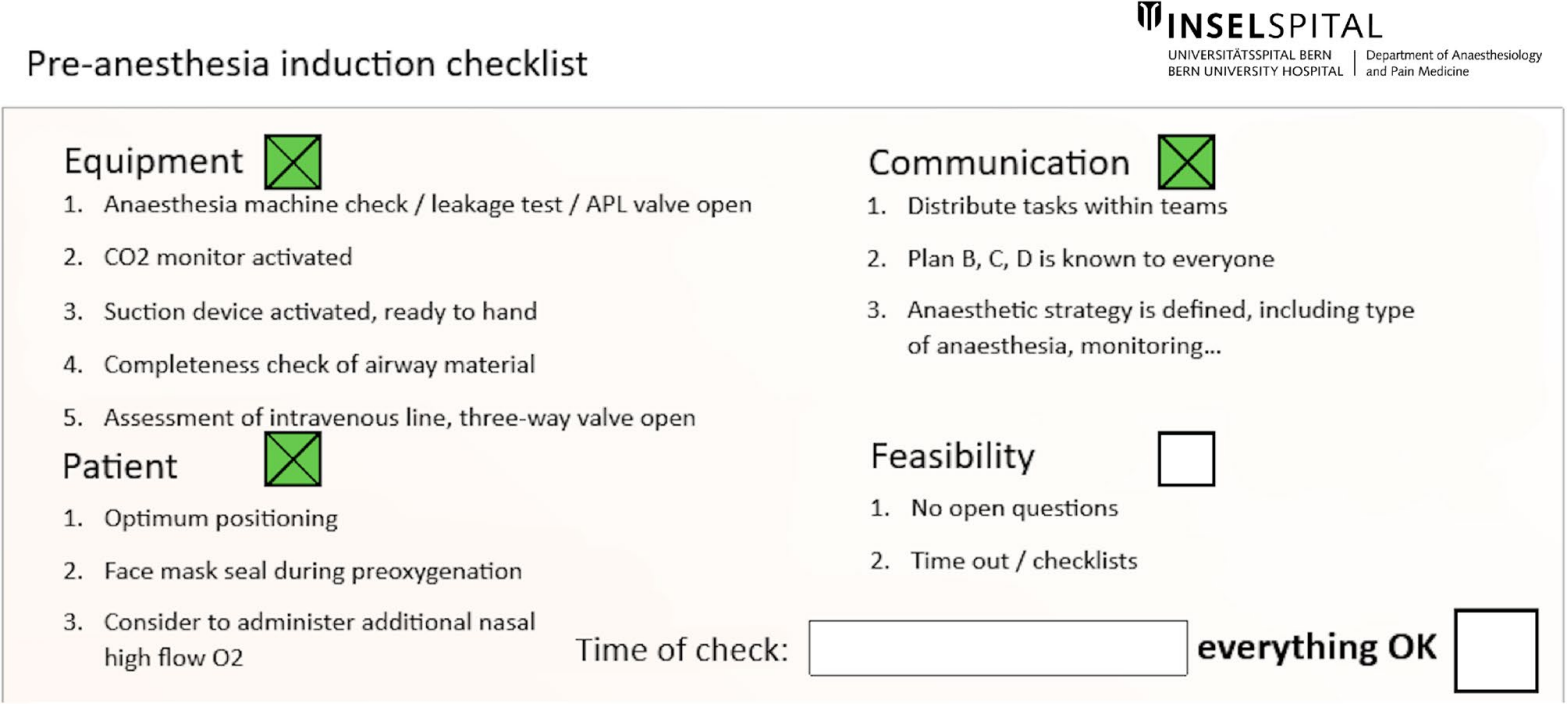
The airway checklist applied at the Bern University Hospital. This checklist should be filled before airway management and induction of anesthesia, for every patient

Sharing the airway management plan with the team, using a preoperative checklist, and verbalizing actions may help reduce avoidable errors or equipment failures [6]. A bundle checklist for difficult airway has been proposed by the PeDI Collaborative[7] and focuses on (i) planning (who will attempt to intubate); (ii) how anesthesia will be induced; (iii) how the patient will be intubated; (iv) a rescue plan; and (v) the “right attitude” (if anyone has a concern, he/she should speak up). Such a simple bundle should be applied in case of planned or unexpected difficult intubation and adapted according to the local availability of expertise and equipment. Another checklist that could be taken as an example is that used at the University Hospital of Bern and presented in Fig. 1.

#### Induction of anesthesia/sedation for airway management

Two recommendations of the recent guidelines [1, 2] are dedicated to pharmacology: (i) “provide adequate level of anesthesia or sedation during airway management” and (ii) “administer a neuromuscular blocking agent to facilitate intubation if spontaneous breathing is not needed”. The authors highlighted the importance of pharmacological treatment during airway management, especially of neonates and infants, to minimize the practice of elective awake intubation. Awake intubation has been routine for many years, especially in non-anesthesia environments. In addition to the psychologic stress for the patient, the consequences of absent or insufficient sedation are well identified: increased intracranial and blood pressure and risk of intracranial hemorrhage [8]. Moreover, intubation of an awake neonate or infant is often more difficult than if it is performed under sedation, especially if the operator is not skilled: the patient is indeed moving and resisting. The PRETTINEO research group focused on the long-term neurocognitive consequences of neonatal intubation, especially looking at the pharmacological treatment during intubation and the need to ensure physiologic stability to minimize systemic hypertension or hypotension. [8] Irrespective of the induction strategy chosen, the frequency of prolonged desaturation did not differ significantly between atropine used with propofol or atropine used with atracurium and sufentanil and was over 60%.

In the operating room, inhalation induction with a volatile anesthetic, such as sevoflurane via face mask, is most often used. Inhalation induction is smooth, can be titrated easily according to the child's response, and facilitates intravenous access. In certain conditions and when the competence is adequate, an intravenous induction of sedation or general anesthesia can be chosen. Awake airway management should be limited to selected cases or acute life-threatening situations (e.g., neonatal resuscitation or cardiac arrest). The PeDI-Collaborative recently published a report from the registry on awake airway management. Children with a high risk of airway obstruction, for example, Pierre Robin Syndrome, benefits from the awake insertion of a supraglottic device followed by tracheal intubation under general anesthesia. Sedation or general anesthesia should be carefully titrated in this category of patients since the administration of sedatives can easily lead to airway obstruction and hypoxemia [9]. Using a nasopharyngeal or supraglottic airway improves the child’s oxygenation, ventilation, comfort, and the conditions for intubation with advanced equipment like a video laryngoscope or fiberoptic. Similarly, inserting a supraglottic device can improve ventilation when face mask ventilation is likely to fail.

The evidence regarding the use of neuromuscular blocking agents (NMBA) prior to tracheal intubation when spontaneous ventilation is not necessary comes mostly from cohort studies. PeDI collaborative provides evidence that difficult or impossible face mask ventilation can be improved by the administration of an NMBA agent [10], especially if the cause of the difficulty is functional (i.e., laryngospasm).

The most widely used NMBA is rocuronium. It has a rapid onset and allows rapid reversal with sugammadex if intubation fails. An alternative to achieve good intubation conditions is remifentanil combined with propofol [11]. Ono et al. showed that 3 mcg/kg of remifentanil is required to achieve good conditions for intubation. However, the potential hemodynamic side effects (bradycardia) as well as the risk of so-called thoracic rigidity (in fact glottis closure) should be considered when this strategy is chosen. The adjunct of topical anesthesia with lidocaine can reduce the ED50 required for propofol and remifentanil. The most frequently described method of topical anesthesia is to spray lidocaine (maximum dose 4 mg/kg) onto the laryngeal and tracheal mucosa under direct vision, meaning that a longer or supplemental laryngoscopy is needed [12]. Other techniques, like lidocaine nebulization or applying some lidocaine jelly on the outside of the tracheal tube, are described but are less effective [12].

#### Preoxygenation, apneic oxygenation, and “per-oxygenation”

Traditional pre-oxygenation can be challenging in children due to a lack of compliance or collaboration. Children, especially young ones, might not cooperate with placing a face mask or feel anxious. However, administration of supplemental oxygen before induction of anesthesia and throughout the entire intubation time is an essential component to minimize the risk of desaturation and extend the so-called safe apnea time, which is defined as the time during which S*p*O_2_ does not decrease below 90% in non-cyanotic patients. In this context, the concept of “per-oxygenation” becomes particularly relevant. Unlike pre-oxygenation, which involves breathing high concentrations of oxygen prior to a procedure to maximize oxygen stores in the body, “per-oxygenation” refers to the continuous administration of oxygen during periods of apnea or spontaneous ventilation until airway management is concluded. This continuous oxygen supply aims to prevent desaturation by maintaining elevated levels of oxygen in the alveoli, thereby supporting ongoing gas exchange whether the patient is breathing or not. Understanding and utilizing “per-oxygenation” techniques can significantly improve the management of patients in whom pre-oxygenation is not possible or inefficient. Apneic oxygenation applies the principle of a ventilatory mass flow, which relies on the continuous flow of oxygen into the airways even without active breathing. Regardless of the flow administered, it creates a reservoir of oxygen within the nasopharynx and trachea, which passively diffuses into the alveoli. The natural consumption of oxygen lowers the alveolar oxygen pressure, creating a gradient that promotes oxygen flow from the upper airway down to the alveoli [13], in addition to the small movements induced by the cardiac oscillations. For this process to occur effectively, the airway must remain open, as any obstruction can impede the flow to the alveoli. As a result, even without mechanical ventilation, the oxygen levels in the blood can be maintained for a longer time, thereby reducing the risk of hypoxemia during procedures such as intubation. Apneic oxygenation contributes to higher first attempt intubation success and extends the safe apnea time, thus increasing the time available for airway management, ultimately contributing to decreased operator stress [14].

Various techniques have been described for improving oxygen delivery. First, the interface used can vary: nasal cannulae, a nasopharyngeal/oral airway or the laryngoscope can deliver oxygen and an inhaled anesthetic during laryngoscopy while the patient is apneic or spontaneously breathing. Second, both low-flow and high-flow oxygen delivery systems have shown similar effectiveness. Low-flow oxygen means delivering oxygen at flows between 0.2 and 1 L/kg/min and can be administered using standard nasal cannulas or a nasopharyngeal airway. The advantages of low-flow systems are simplicity and widespread availability in various clinical settings. High-flow oxygen is the application of warm and humidified oxygen with a flow of 2 L/kg/min to better preserve mucosal function in the respiratory tract. It requires a more sophisticated delivery system. Clinicians should use the technique and device with which they are the most familiar but always consider delivering oxygen throughout the entire airway manipulation, from induction of anesthesia until the airway is secured. Table 3 summarizes oxygen administration techniques with relative tips and risks, while in the supplemental materials, we provide two videos demonstrating the application of low- or high-flow (supplemental 1 and supplemental 2, respectively) oxygen during intubation.

**Table 3:** Oxygen administration techniques, tips and potential risks

Interface	Technique	Oxygen	Tips
Nasal trumpet or nasopharyngeal tube	Connect to the anesthesia circuit	6 L/min with APL valve fully open	Equipment easily available in the operating room
Low-flow nasal cannulae	Connect to extra source of oxygen	0.2–1 L/kg/min	Need for dedicated cannulae and equipment
High-flow nasal cannulae	Connect to high-flow system	2 L/kg/min max 20 L/min	Need for dedicated cannulae and equipment for humidified oxygen
Oral RAE tube	Connect to extra source of oxygen	6 L/min or more	In the corner of the mouth

The recently published second report from the PeDI registry demonstrates a significant reduction of complications compared to the initial cohort. One reason for this reduced incidence could be related to the widespread implementation of per-oxygenation across the participating centers. The PeDI publication incorporated a bundle for the use of oxygen during intubation. Such an implementation of the bundle could be adopted worldwide to remind physicians that oxygen can be easily applied and is safe [7, 15].

#### Airway management

The recent guidelines advocate for utilizing video laryngoscopy (VL) as the preferred method for neonatal intubation [2]. Historically direct laryngoscopy has been used as the technique of choice for tracheal intubation, but the introduction of VL has changed practice for several reasons. When VL with a standard blade, either straight (Miller) or curved (Macintosh), is used, the technique of laryngoscopy is the same as for DL: the blade is introduced in the corner of the mouth to displace the tongue and its tip placed in the vallecula (see accompanying video) before lifting the handle of the laryngoscope obliquely [16, 17]. The screen can be used for teaching when the person in charge of intubation is a novice in DL. Furthermore, in case of difficult laryngoscopy or intubation, the team helping with external manipulations or additional equipment directly visualizes the result of its action.

At least three large randomized controlled trials (RCTs) favor the use of VL with a standard blade vs DL in young patients undergoing tracheal intubation in the operating room, specifically neonates and infants up to 6.5 kg [16–18]. The recently published RCT confirms the favorable use of VL in the neonatal intensive care unit setting [18, 19]. Based on the current evidence, the use of VL with a standard blade is strongly recommended for tracheal intubation in infants and neonates, either in the OR, delivery room, or NICU or if the patient presents a difficult airway. However, all these evidences are based on the use of standard blades in children with normal airway. On the contrary, it is still not fully elucidated when hyperangulated blades should be used [20, 21]. The use of a non-standard blade, whether channeled or not, requires some training indeed because the technique of laryngoscopy is different: the blade is introduced in the middle of the mouth, and the handle is directed vertically when the tip of the blade is positioned in the vallecula. A hyperangulated blade may be a backup strategy in case of poor glottis visualization. The tube needs to be prepared with a stylet or introducer to align with the shape of the blade. A hyperangulated blade can also be used in combination with flexible bronchoscopy (four hands technique), when difficult intubation is expected [22].

### Cannot intubate, cannot oxygenate

The cannot intubate, cannot oxygenate (CICO) situation is a rare but catastrophic event in children. It can lead to rapid desaturation, ending in cardiac arrest, cerebral damage, or death. It was the cause of 20/186 (11%) cardiac arrests reported in the Wake Up Safe Quality improvement initiative [23]. The aims of the careful preoperative assessment of the airway and pediatric difficult airway management guidelines and algorithms are to prevent CICO situations and provide guidance for rescue maneuvers. In the case of a CICO situation, waking the patients and postponing anesthesia and tracheal intubation is often no option, considering the rapid and devastating consequences of prolonged severe hypoxemia. Thus, emergency front-of-neck access (eFONA) [1, 24, 25] is the only option left. NAP4 [26] showed that in adult patients, only 43% of the percutaneous eFONA was successful, in contrast to the surgical techniques, which had an almost 100% success rate. Similarly, the surgical technique should also be preferred in children: in children ≥ 8 years, a surgical cricothyroidotomy with an age-adapted bougie and a tracheal tube should be performed, while in those < 8 years, a tracheostomy between the first and the second or the second and the third tracheal rings, should be performed [27].

For a considerable time, transcutaneous needle cricothyroidotomy has been the recommended eFONA method for infants and children under eight years of age [27]. This preference assumed that anesthesiologists and non-surgical specialists are more reluctant to perform surgical procedures due to limited surgical skills and are more familiar with the Seldinger technique, as used during central venous catheter placement.

It is essential to consider the anatomical features of children when performing a tracheostomy. According to Navsa et al. [28], the mean dimensions of the cricoid membrane in neonates are 2.6 mm in height and 3.0 mm in length, indicating that a tracheal tube with an outer diameter exceeding 2.5 mm is too wide and may cause damage to the surrounding structures. This poses a challenge in implementing the recommendations for cricothyrotomy, especially in infants. Although CICO scenarios and the necessity for eFONA are rare, airway practitioners must be prepared for this situation.

In the absence of definitive human data, which are difficult to obtain, the synthesis from the three studies performed in animal models shows that the scalpel bougie tracheostomy is associated with a higher success rate and significantly fewer injured tracheal rings [24, 29, 30]. The use of a Frova Intubating Introducer (Cook Medical, Bloomington, USA) not only facilitates a safe tracheal tube insertion but also allows initial oxygenation through the catheter using devices based on the Bernoulli principle and its variant, the Venturi effect, like the Ventrain^®^ (Medicalsol, Switzerland). The major advantages of Ventrain are active expiration and the need for a very low ventilation rate with an inspiratory and expiratory ratio of 1.

Consistent training should be accessible to all staff members who may encounter a CICO situation, regardless of their medical specialization or expertise level [29]. Given the steep learning curve observed during the initial four attempts, a minimum of four to five consecutive eFONA procedures should be performed on a rabbit’s trachea during each training session [29]. Currently, there is little evidence-based guidance on the optimal frequency of such training sessions to maintain practical skills, but brush-up training every three months is suggested [31]. Training should not only solely focus on the technical execution of the eFONA technique but also include comprehensive team-based training covering advanced planning, communication, decision-making, and procedural efficacy, addressing various aspects to mitigate the impact of human factors. For example, national and international conferences could consider spaces for workshops and allow attendees to practice eFONA techniques.

**Fig. 2:**
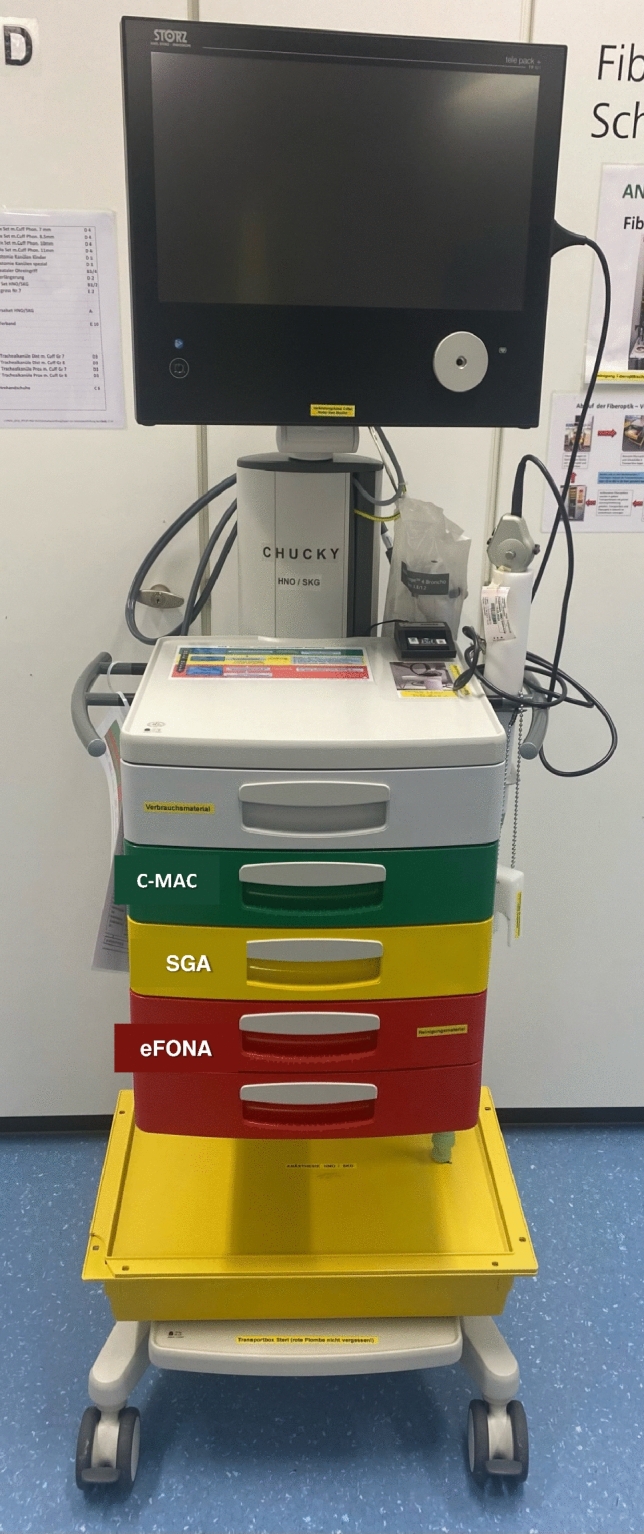
Example of the difficult airway trolley at Bern University Hospital, mirroring the cognitive aids

The prompt availability of airway equipment is crucial. After choosing an emergency airway approach based on local needs, equipment, and trained skills, an emergency tracheotomy set containing standardized equipment should be readily available. Clinicians should be regularly trained in its use, aware of its location, and routinely review its content. Figure 2 shows an example of the trolley used in the University Hospital of Bern.

### Extubation

While induction and intubation of children need particular attention, so does extubation. Extubation is never an emergency and should be appropriately prepared, which means having a re-intubation plan and strategies to manage any immediate post-extubation complications. In reinforcing the concept that extubation is never an emergency, the aim is to prioritize the child's safety and comfort. By avoiding rush and improvisation, healthcare providers ensure that the transition from mechanical ventilation to natural breathing proceeds with a natural airway as smoothly and predictably as possible. Before extubation, the airway practitioners should remember the medical history of the child, whether intubation was difficult, either expected or unexpected, and conduct a detailed assessment to ensure the child is ready for extubation, which includes verifying the absence of any residual neuromuscular blockade (monitoring and clinical signs) if a muscle relaxant has been used, checking that the child can maintain effective spontaneous breathing with sufficient upper airway reflexes to protect its airway post-extubation [32]. The same equipment and expertise used for intubation should be ready during extubation. Weatherall et al. proposed the so-called R2D2 (Risk, Ready, Do, Discharge) approach for extubation [33]. A child should be evaluated regarding the preoperative risk (R) of difficult airway, evaluated if ready (R) for extubation, execute the extubation maneuver (D) in the safest location during the correct time of the day, and discharge (D) to the correct location, ward or Pediatric Intensive Care Unit (PICU), with a re-intubation plan in place. This systematic approach, accompanied by cognitive aids, can be easily learned and applied to every extubation setting, especially if adverse events complicated the intubation [34]. A child who is at high risk of post-extubation failure could receive some treatments to reduce the risk. Applying high-flow nasal oxygen might support a borderline respiratory distress condition in the early post-extubation period [35]. In contrast, a post-extubation stridor can be treated with nebulized adrenaline and or steroids [2].

## Addressing human factors in pediatric airway management

Analysis of airway-associated adverse events taught us that human factors are as important as technical skills since they significantly affect personal and team performance and, therefore, patient outcomes and safety [36]. For this reason, concerns about human factors were introduced in the 2015 Difficult Airway Society Guidelines for adults [37] and reinforced in the 2023 BJA and ESAIC joint guidelines for neonatal airway management [2]. Like other human beings, anesthesiologists may have physical and cognitive limitations regarding their workload capacity and the amount of information they can process in a stressful situation. Moreover, anesthesiologists often deal with ever-changing team compositions, high time pressure and workload, and fatigue, especially during night shifts [26].

Several cognitive aids and systems aimed at preventing critical events exist, such as checklists (Fig. 1), uniformly color-coded medication and airway trolleys [38], and algorithms for critical situations. Graphic elements and color codes are useful because they can speed up the search for the necessary device and avoid errors [38]. The authors of the “Vortex approach” conceived a high-acuity implementation tool to support the multidisciplinary team involved in an airway crisis [39]. The Vortex tool provides a well-defined framework for designing the trolley, ensuring a balance between clutter and the inclusion of all possible alternatives for various situations. It also incorporates a comprehensive array of labels for drawers’ identification, enhancing the familiarity and intuitiveness of tool selection. Figure 2 represents how the trolley is labeled at Bern University Hospital, mirroring the cognitive aids.

Bad or inadequate communication is one of the most important factors leading to patient harm [40] and should be avoided. Communication should be bidirectional or “closed loop” [41] to prevent misunderstandings, meaning both sides confirm receiving the information [42, 43]. Another important point of high-performing teams is to ensure psychological safety. It encourages open communication and supports team members in expressing their concerns or ambiguities [43] regardless of their role or hierarchic position, without fearing negative comments or consequences. This is even more efficient if team leaders and senior staff are trained to “speak up” if a safety concern arises and to “listen down” for key phrases [43]. Communication also seems to improve if the team members often work together since they get to know each other’s strengths and limitations. However, it isn't easy in large departments to always have the same teams working together. It is also important to organize regular simulations of difficult situations to improve individual and team performance [43].

Critical events must be analyzed constructively during a benevolent debriefing to avoid reoccurrence. Moreover, psychological support for the team exposed to the event is mandatory to prevent post-traumatic stress. But noncritical situations should be debriefed, too, and the team discussion should focus on what worked well in a process so that the team can learn from cases that evolved without adverse events and from cases that evolved differently from the initial plan but without adverse events. A 2022 online survey sent to all members of the Society for Pediatric Anesthesia in the USA analyzed the following topics: debriefing utility, use, impact, time provided, practice setting, perceived impact, and hurdles to participate. Among the 294 responders, around 54% reported having participated in a short debriefing, 39% found the debriefing useful for root-cause analysis, and 54% found it helpful for processing the event emotionally. However, for over 90% of them, the lack of time was the most significant limiting factor for not attending the debriefing, followed by the availability of facilitators for debriefings (59%). This study shows that providing enough time for a debriefing and specialized personnel is essential for the staff to process these critical events and may lead indirectly to better patient safety/outcome [44].

To keep the performances high, minimize the incidence of peri-intubation complications, train trainees and registrars, introducing specific pediatric anesthesia training in their curriculums [45] is imperative. For example, in Scandinavia, a pediatric curriculum for specialist anesthesiologists was set up in 2003. The aim was to improve skills in pediatric anesthesia and provide a certification. Candidates usually spend 12 months in a Children's hospital, between the pediatric intensive care unit (3 months) and pediatric operating rooms (9 months). An assigned tutor ensures they perform enough cases and acquire professional competencies during this period. In addition, there are three courses of 4 days each, including topics like ventilatory support, difficult airway management, and regional anesthesia, which are discussed with experienced pediatric anesthetists [45]. The Scandinavian approach can be an example of setting up a pediatric curriculum and could be totally or partially applied in other countries. Once the certification of a pediatric anesthetist is obtained, regular exposure to pediatric cases of all ages is mandatory to maintain competencies and keep performances high.

## Conclusions

In pediatric airway management, achieving efficiency and safety is a challenging yet essential goal. The unique anatomical and physiological characteristics of pediatric patients necessitate specialized knowledge, skills, and equipment. Healthcare providers must stay updated with the latest techniques, technologies, and guidelines to ensure the highest standard of care.

Continuous education and hands-on training are critical components in improving outcomes in pediatric airway management. Simulation-based training and regular drills can enhance practitioners' proficiency and confidence. Moreover, incorporating multidisciplinary teamwork ensures comprehensive and coordinated care, further reducing the risk of complications.

**Fig. 3:**
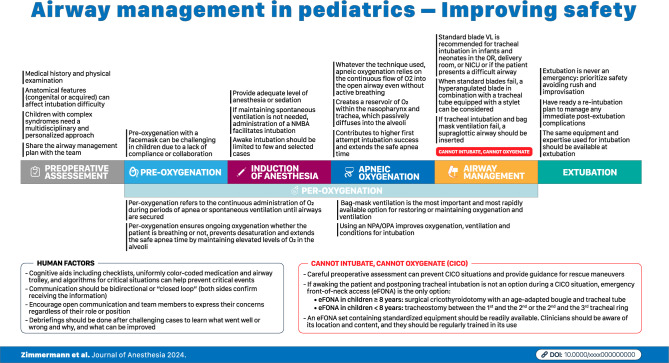
Infographics representing the most relevant steps for pediatric airway management

The journey toward perfection in pediatric airway management is ongoing. By prioritizing patient safety, embracing innovation, and fostering a culture of continuous improvement, healthcare providers can significantly improve the quality of care (Fig. 3). Ultimately, the road to perfection is paved with dedication, collaboration, and a commitment to the well-being of our youngest and most vulnerable patients.

## Supplementary Information

Below is the link to the electronic supplementary material.

Supplementary file1 (MP4 255941 KB)

Supplementary file2 (MP4 261109 KB)
